# Prevention of Tracheo-Innominate Artery Fistula by Using an Adjustable Tracheostomy Tube

**DOI:** 10.7759/cureus.20043

**Published:** 2021-11-30

**Authors:** Koji Obara

**Affiliations:** 1 Neurology, National Hospital Organization Akita National Hospital, Yurihonjo, JPN

**Keywords:** tracheo-innominate artery fistula, tracheostomy tube, retrocollis, parkinsonism, multiple system atrophy

## Abstract

Tracheo-innominate artery fistula (TIF) is a severe complication associated with a long-term tracheostomy, and TIF-associated bleeding has a high mortality rate. Here, we report two patients who were considered to be at high risk of developing TIF due to retrocollis after tracheostomy. The patients were an 82-year-old woman with Parkinson’s disease (PD) and a 64-year-old man with multiple system atrophy (MSA). Both patients underwent tracheostomy at an advanced stage and later showed retrocollis. Colored and 3D-reconstructed computed tomography (CT) showed tracheal deformation into a C curve, with the tip of the tracheostomy tube attached to the anterior wall, where the innominate artery transverses. Since they were considered to be at high risk of developing TIF, we used an adjustable tracheostomy tube. Follow-up CT revealed that the tip of the new tracheostomy tube had separated from the tracheal anterior wall. Although retrocollis is rare in PD and MSA, it can develop at the end stage of these diseases. An adjustable tracheostomy tube may be an option for preventing TIF development in cases where surgical intervention would be difficult.

## Introduction

Tracheo-innominate artery fistula (TIF) is a rare but life-threatening complication of tracheostomy [1-3]. Factors such as low tracheostomy, over-inflated cuffs, long-standing tracheostomy, and neck/chest deformity are reported to contribute to TIF formation [2,3]. Here, we report two patients at high risk of TIF who developed retrocollis after tracheostomy due to Parkinson’s disease (PD) and multiple system atrophy (MSA).

## Case presentation

Case 1

**Figure 1:**
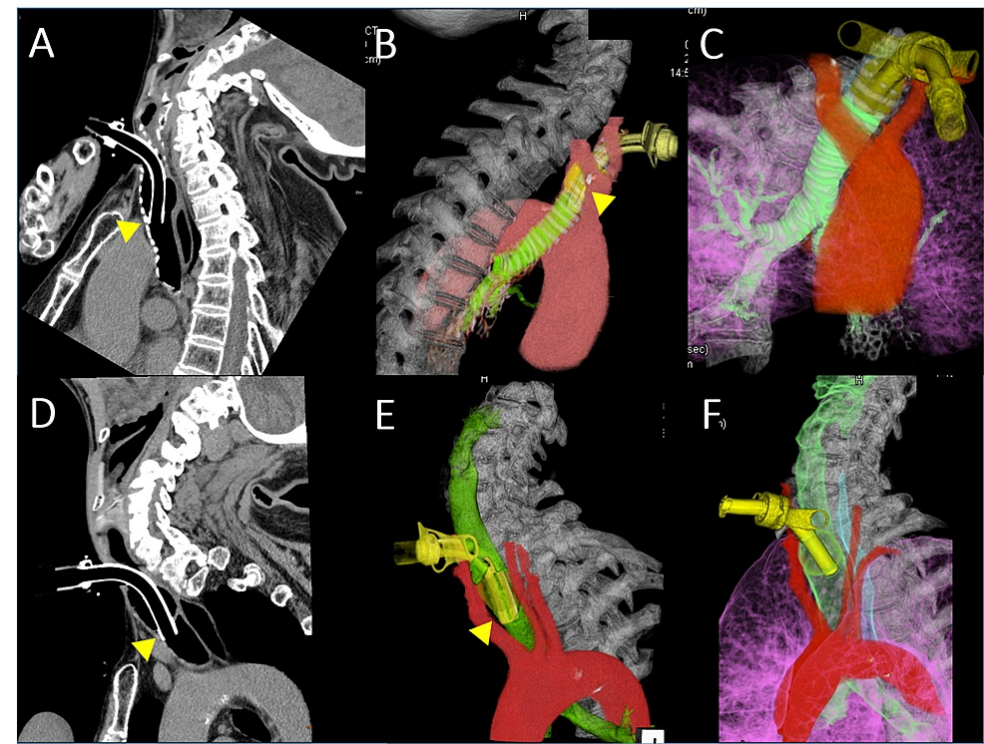
Neck-thoracic CT Computed tomography findings. (A-C) Case 1 with Parkinson’s disease. (A) The neck is in a severe extended position (retrocollis). (B) Colored and 3D-reconstructed images show that the tip of an ordinary silicone tracheostomy tube has attached to the tracheal anterior wall, where the innominate artery transverses (arrowhead). (C) After switching to an adjustable tracheostomy tube, CT shows that the tip of the adjustable tracheostomy tube is in a safe position away from the tracheal anterior wall. (D-F) Case 2 with multiple system atrophy. (D) Case 2 also shows severe retrocollis. (E) The tip of an ordinary silicone tracheostomy tube has attached to the tracheal anterior wall across the innominate artery (arrowhead). (F) After switching to an adjustable tracheostomy tube, the tip of the adjustable tracheostomy tube is confirmed to be in a safe position.

The patient was an 82-year-old woman with an onset of Parkinsonism including bradykinesia, hand tremor, and limb rigidity at the age of 57 years. She was diagnosed with PD at that time. In the years following onset, her symptoms improved with levodopa, but motor fluctuation subsequently became apparent. At the age of 73, she was admitted to our hospital. On neurological examination, she was alert and oriented. She had no gaze limitation. She showed bradykinesia, bilateral resting hand tremor, and limb rigidity without laterality. Although she had severe postural instability, she could walk slowly by taking small steps using a walker. Muscle stretch reflexes were normally elicited, and the plantar response was flexor bilaterally. Laboratory examination was unremarkable. Brain magnetic resonance imaging (MRI) showed no remarkable findings. ^123^I-meta-iodobenzylguanidine (^123^I-MIBG) myocardial scintigraphy showed 1.18 (normal ratio 2.1 or higher) at the early heart-to-mediastinum ratio. Subsequently, she became akinetic and bedridden. She had never been administered neuroleptics. She underwent gastrostomy due to severe dysphagia at the age of 75 years, and tracheostomy at the age of 80 years due to recurrent aspiration pneumonia. After tracheostomy, she gradually developed severe retrocollis (Figure 1A). Colored and 3D-reconstructed neck-thoracic computed tomography (CT) revealed that the tip of the tracheostomy tube had attached to the tracheal anterior wall where the innominate artery transverses (Figure 1B). We changed her tracheostomy tube from an ordinary silicone tube to an adjustable tracheostomy tube (GB Adjustfit Tracheostomy Tube; Fuji Systems, Tokyo, Japan). Follow-up CT revealed that the tip of the adjustable tracheostomy tube had separated from the tracheal anterior wall (Figure 1C).

Case 2

The patient was a 64-year-old man with an onset of ataxic gait at the age of 54 years. He showed progressive gait disturbance and dysarthria, with subsequent autonomic failure involving urinary incontinence and orthostatic hypotension. At the age of 58 years, he became bedridden and was admitted to our hospital. On neurological examination, he was alert and oriented but had difficulty conveying his wishes due to severe dysarthria. He had no gaze limitation and was nystagmic but saccadic. He showed bradykinesia and dysmetria and oscillation in limb movements. Muscle stretch reflexes were normally elicited, and the plantar response was flexor bilaterally. Laboratory examination was unremarkable. Brain MRI revealed marked atrophy of the cerebellum and the pons. The hot cross bun high-signal sign on T2-weighted imaging was seen in the central pons. He was diagnosed as probable MSA with predominant cerebellar ataxia. He was treated with levodopa and taltirelin without apparent improvement. He had never been administered neuroleptics. Due to recurrent aspiration and respiratory failure, he underwent tracheostomy at the age of 61 years, followed by mechanical ventilation. After tracheostomy, he gradually developed limb rigidity and severe retrocollis (Figure 1D). Colored and 3D-reconstructed neck-thoracic CT revealed that the tip of the tracheostomy tube had attached to the tracheal anterior wall where the innominate artery transverses (Figure 1E). Similarly, we changed his tracheostomy tube to the same adjustable tracheostomy tube as in Case 1. Follow-up CT revealed that the tip of the adjustable tracheostomy tube had separated from the tracheal anterior wall (Figure 1F).

## Discussion

TIF is a severe complication associated with long-term tracheostomy [1-3]. Once TIF bleeding occurs, it leads to massive life-threatening hemorrhage and airway obstruction [1-3]. Without prompt surgical intervention, this complication is almost always fatal [1]. For this reason, the prevention of TIF is crucial.

Neck deformity is one of the contributing factors to TIF formation [2,4]. Our two patients with PD and MSA showed a neck extension deformity, so-called retrocollis, after tracheostomy. On colored and 3D-reconstructed CT without using a contrast agent, we were able to clearly delineate the positional relationship of the trachea, the arteries including the innominate artery, and the tracheostomy tube. It was found that, due to retrocollis, the trachea was deformed into a C curve and the tip of the tracheostomy tube had attached to the tracheal anterior wall, where the innominate artery transverses. The relationship between tracheal anterior wall compression by the tip of the tracheostomy tube and TIF formation has previously been reported [4-6]. From our CT findings and evidence from the literature, our patients were considered to be at high risk of developing TIF. The most effective diagnostic tool for TIF is bronchoscopy, which can directly observe erosion and pulsation of the tracheal anterior wall [7-9]. In contrast, colored and 3D-reconstructed CT can promptly demonstrate a digital image of the structure surrounding the trachea. Moreover, since a contrast agent is not always necessary due to colored reconstruction, the burden on the patient is very small [5].

**Figure 2:**
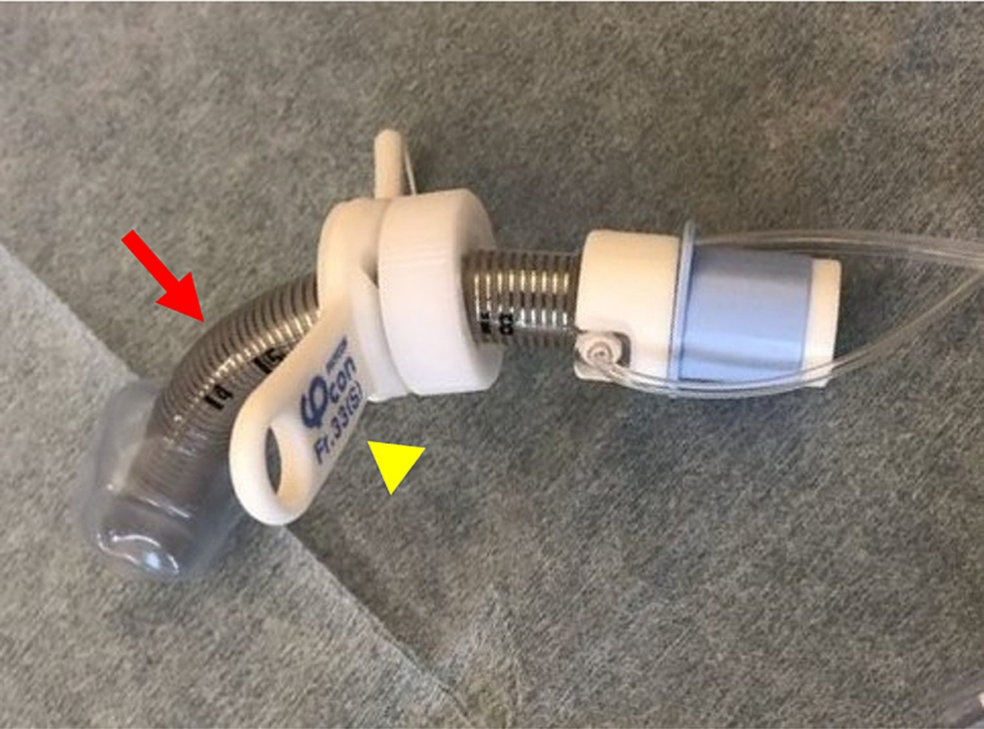
Adjustable tracheostomy tube Movable wing (arrowhead). A flexible tube with spiral stainless-steel wire (arrow).

The best treatment for TIF is its prevention and proper tracheostomy care plays a critical role [6,10]. This includes appropriate placement of a tracheostomy tube, its size and material, hygiene, and avoidance of prolonged over-inflation of the cuff [4,11]. Fundamental preventive measures for TIF formation include procedures such as innominate artery ligation [3]. However, medical resource limitations at our institution rendered such procedures difficult. Instead, we chose to switch from an ordinary silicone tube to an adjustable tracheostomy tube, which has already been used in patients with tracheal deformities due to severe motor and intellectual disabilities [12]. The spiral stainless-steel wire inside the wall of the tube provides flexibility and fits the shape of the trachea, while the movable wing allows us to adjust the depth of intubation (cuff) (Figure 2). We manually adjusted the depth and the curvature of the adjustable tracheostomy tube with reference to the CT image. On follow-up CT, we confirmed that the tip of the new tube was in a safe position away from the tracheal anterior wall. Adjustable tracheostomy tubes are more expensive than ordinary silicone tubes [11]. First, it should be considered if the risk of TIF can be reduced by applying a piece of gauze around the stoma to pull an ordinary tracheostomy tube outside. However, since an ordinary tracheostomy tube has a fixed curvature, the curvature must be individually customized for a patient whose trachea is severely deformed [13,14]. Hence, an adjustable tracheostomy tube may be more suitable for patients with severe and progressive retrocollis like our cases.

Retrocollis is often seen in patients with progressive supranuclear palsy (PSP), especially at the advanced stage [15,16]. In contrast, in patients with PD and MSA, anterocollis is often reported, but retrocollis is rare [16,17]. However, Kashihara et al. has reported that retrocollis is not uncommon in patients with end-stage PD and in patients with end-stage PD and MSA, whose anterocollis reverses to retrocollis as the disease progresses [16,17]. Similarly, our two cases with PD and MSA slowly presented with retrocollis after tracheostomy was performed at the advanced stage of the disease. Taken together, in patients with parkinsonism such as PSP, PD, and MSA who have undergone tracheostomy, cervical posture, and positional relationships among the trachea, innominate artery, and tube should be regularly observed.

## Conclusions

We used an adjustable tracheostomy tube to prevent TIF in patients with retrocollis. Retrocollis can appear at the end stages of PD and MSA. Our cases indicate that an adjustable tracheostomy tube may be an alternative option for preventing TIF in cases in which surgical intervention is challenging. Further case accumulation and long-term observation should be conducted to provide strong evidence for the usage of an adjustable tracheostomy tube in such cases.
